# Effects of Sarcolemmal Background Ca^2+^ Entry and Sarcoplasmic Ca^2+^ Leak Currents on Electrophysiology and Ca^2+^ Transients in Human Ventricular Cardiomyocytes: A Computational Comparison

**DOI:** 10.3389/fphys.2022.916278

**Published:** 2022-06-16

**Authors:** Molly E. Streiff, Frank B. Sachse

**Affiliations:** ^1^ Nora Eccles Harrison Cardiovascular Research and Training Institute, University of Utah, Salt Lake City, UT, United States; ^2^ Department of Biomedical Engineering, University of Utah, Salt Lake City, UT, United States

**Keywords:** calcium, cardiomyocyte, sarcolemma, sarcoplasmic reticulum, leak

## Abstract

The intricate regulation of the compartmental Ca^2+^ concentrations in cardiomyocytes is critical for electrophysiology, excitation-contraction coupling, and other signaling pathways. Research into the complex signaling pathways is motivated by cardiac pathologies including arrhythmia and maladaptive myocyte remodeling, which result from Ca^2+^ dysregulation. Of interest to this investigation are two types of Ca^2+^ currents in cardiomyocytes: 1) background Ca^2+^ entry, i.e., Ca^2+^ transport across the sarcolemma from the extracellular space into the cytosol, and 2) Ca^2+^ leak from the sarcoplasmic reticulum (SR) across the SR membrane into the cytosol. Candidates for the ion channels underlying background Ca^2+^ entry and SR Ca^2+^ leak channels include members of the mechano-modulated transient receptor potential (TRP) family. We used a mathematical model of a human ventricular myocyte to analyze the individual contributions of background Ca^2+^ entry and SR Ca^2+^ leak to the modulation of Ca^2+^ transients and SR Ca^2+^ load at rest and during action potentials. Background Ca^2+^ entry exhibited a positive relationship with both [Ca^2+^]_i_ and [Ca^2+^]_SR_. Modulating SR Ca^2+^ leak had opposite effects of background Ca^2+^ entry. Effects of SR Ca^2+^ leak on Ca^2+^ were particularly pronounced at lower pacing frequency. In contrast to the pronounced effects of background and leak Ca^2+^ currents on Ca^2+^ concentrations, the effects on cellular electrophysiology were marginal. Our studies provide quantitative insights into the differential modulation of compartmental Ca^2+^ concentrations by the background and leak Ca^2+^ currents. Furthermore, our studies support the hypothesis that TRP channels play a role in strain-modulation of cardiac contractility. In summary, our investigations shed light on the physiological effects of the background and leak Ca^2+^ currents and their contribution to the development of disease caused by Ca^2+^ dysregulation.

## Introduction

Ca^2+^ concentrations are dynamically controlled in cardiomyocytes by a complex regulatory system comprising ion channels, transporters, exchangers, regulatory proteins, and ion buffers. Intricately regulated levels of Ca^2+^ concentrations are critical for electrical activity, excitation-contraction coupling, and other signaling pathways. In many cardiac diseases, the delicate balance of Ca^2+^ cycling is perturbed. Ca^2+^ dysregulation underlies maladaptive cardiac remodeling. A complete understanding of all components of Ca^2+^ handling is essential for the development of therapeutic strategies to attenuate cardiac pathologies.

Many ion channels underlying the Ca^2+^ signaling in cardiomyocytes are well characterized. Ca^2+^ signaling related to excitation-contraction coupling primarily relies on sarcolemmal Ca^2+^ entry through voltage-gated L-type Ca^2+^ channels (LTCC) to trigger Ca^2+^-induced Ca^2+^ release from the sarcoplasmic reticulum (SR) through ryanodine receptors (RyR), and, subsequently, Ca^2+^ extrusion *via* the sodium-calcium exchanger (NCX) and reuptake into the SR through sarco/endoplasmic Ca^2+^-ATPase (SERCA). Other Ca^2+^ currents contribute to the modulation of Ca^2+^ concentrations in cardiomyocytes as well. Of interest to this study are background Ca^2+^ entry through the sarcolemma and SR Ca^2+^ leak. Understanding of the physiological role of these Ca^2+^ currents is still incomplete.

Beyond Ca^2+^ transport across the sarcolemma from the extracellular space into the cytosol through LTCC and NCX, sarcolemmal Ca^2+^ transport comprises background Ca^2+^ entry. In resting cardiomyocytes, [Ca^2+^]_i_ is of the order of 100 nM, which indicates the existence of a background Ca^2+^ entry pathway to balance the Ca^2+^ efflux of NCX (Eisner et al., 2020). Resting ventricular myocytes depleted of Ca^2+^ stores with caffeine are able to reload the SR by a mechanism that involves extracellular Ca^2+^, demonstrating further evidence of background Ca^2+^ entry (Terracciano and Macleod, 1996). Based on studies measuring background Ca^2+^ influx in rat ventricular myocytes of the order of 2–5 μmol/L per second (Choi et al., 2000) or 4 μmol/L per second (Sankaranarayanan et al., 2017), the background Ca^2+^ entry is approximately 10% of the influx through LTCC current (5–10 μmol/L each action potential) at normal heart rates (Eisner et al., 2020). The identity of background Ca^2+^ flux is still poorly defined, but several channels have been suggested as contributors.

One study identified a Ca^2+^ entry mechanism that is blocked by the nonspecific agent gadolinium (Gd^3+^) (Kupittayanant et al., 2006). Connexin hemichannels are candidates for background Ca^2+^ entry since they can be inhibited by Gd^3+^ (Stout et al., 2002). While connexin hemichannels primarily form pairs to allow ion fluxes between cells at intercalated discs, some are present as hemichannels in the surface membrane of a single cell (Wang et al., 2012; Leybaert et al., 2017) and may, therefore, provide a route for Ca^2+^ entry. However, primary candidates for background Ca^2+^ entry include members of the family of Transient Receptor Potential (TRP) channels, which are also sensitive to Gd^3+^. The *mdx* mouse model of muscular dystrophy exhibits [Ca^2+^]_i_ and [Na^+^]_i_ overload that can be blocked by Gd^3+^, and the increase in cation entry has been suggested to involve TRPC channels (Mijares et al., 2014). Myocytes from old *mdx* mice exhibit increased expression of a putative stretch-activated channel (SAC), TRPC1. Elevated [Ca^2+^]_i_ levels can also be reduced to [Ca^2+^]_i_ levels of WT myocytes when exposed to SAC blockers streptomycin or GsMTx-4 (Williams and Allen, 2007; Ward et al., 2008). Upregulated TRPC1 also contributes to increased [Ca^2+^]_i_ through SAC in hypertrophic myocardium of rats following isoproterenol injection (Chen et al., 2013). Background Ca^2+^ entry involved in maladaptive cardiac remodeling was more recently shown to critically depend on both TRPC1 and TRPC4 (Camacho Londono et al., 2015, Camacho Londoño et al., 2021). TRPC6 channels have also been shown to modulate cytosolic Ca^2+^ transients and SR Ca^2+^ load through sarcolemmal Ca^2+^ entry (Ahmad et al., 2020). Furthermore, TRPV4 can modulate Ca^2+^ transients and SR load, and participates in hypoosmotic stress-induced cardiomyocyte Ca^2+^ entry (Rubinstein et al., 2014; Jones et al., 2019).

Ca^2+^ transport across the SR membrane from the intracellular store into the cytosol is known as SR Ca^2+^ leak. During an action potential, activation of RyR clusters triggered by Ca^2+^ entry through LTCC results in a synchronized release of a large amount of Ca^2+^ from the SR that forms the Ca^2+^ transient and leads to cardiomyocyte contraction. Activation of RyR clusters causes Ca^2+^ sparks. These sparks are an important pathway for SR Ca^2+^ leak. RyR Ca^2+^ leak can occur also through a mechanism independent of sparks (Santiago et al., 2010). Further, total SR Ca^2+^ leak includes a component separate from RyRs (Zima et al., 2010). Many candidates have been suggested as components of this leak, yet it is still ill-defined. A candidate is the inositol 1,4,5-trisphosphate (IP_3_) receptor (IP_3_R), which is a Ca^2+^ release channel expressed at lower densities than RyRs in cardiomyocytes and found upregulated in heart failure (HF) (Go et al., 1995; Ai et al., 2005) and therefore may be a relevant contributor to SR Ca^2+^ leak (Zima et al., 2010). Members of the TRP family have also been suggested to contribute to SR Ca^2+^ leak. TRPC1 was found to operate as a SR Ca^2+^ leak channel in skeletal muscle (Berbey et al., 2009) and more recently in cardiomyocytes (Hu et al., 2020). Additional evidence suggests contribution of TRPC6, TRPM8, TRPP2, and TRPV1 to endoplasmic reticulum Ca^2+^ leak in various cell types, although characterization is still incomplete for cardiomyocytes (Lemos et al., 2021).

TRP channels constitute primary candidates for explaining background and leak Ca^2+^ currents through both the sarcolemma and the SR membrane. Interestingly, many members of the TRP family are known to be modulated by stretch (Inoue et al., 2009; Reed et al., 2014; Peyronnet et al., 2016). Cardiomyocyte contractility is known to respond to mechanical stretch in two phases: a rapid and a slow response (Calaghan and White, 1999). The rapid response is the cellular basis for the Frank-Starling Mechanism (FSM). It relies primarily on myofilament overlap and alteration of myofilament Ca^2+^ sensitivity, and does not involve changes in Ca^2+^ transients. The slow response has been termed slow force response (SFR) or stress-induced slow increase in contractility (SSC), describing the gradual increase in twitch force corresponding to an increase in [Ca^2+^]_i_ transients that develops over several minutes when stretch is sustained. Members of the TRP family are likely candidates for mechanotransduction of the SFR.

Though background and leak Ca^2+^ currents are still poorly defined, they may play important roles in regulating Ca^2+^ homeostasis and contractility and altered Ca^2+^ dynamics in cardiac disease. The lack of complete understanding of the identity and mechanics of these channels, in addition to their relatively small amplitude compared to the voltage-gated Ca^2+^ channels, make them difficult to study *in vivo*. A computational model provides the advantage to study the currents and their roles in isolation from other cellular mechanisms. In this study, we use a mathematical model of a human ventricular myocyte to analyze the individual contributions of background Ca^2+^ entry and SR Ca^2+^ leak to the modulation of Ca^2+^ transients and SR Ca^2+^ load. We also assess the effects of the Ca^2+^ currents on cellular electrophysiology.

## Materials and Methods

### Mathematical Model of Ventricular Myocyte

We applied a mathematical model of a human ventricular myocyte (Grandi et al., 2010). The model and subsequent analyses were executed in MATLAB (R2020b). The model includes subsarcolemmal and junctional compartments beyond the cytosol compartment. Total background Ca^2+^ current (I_Cabk_) through the sarcolemma is defined as a summation of junctional (I_Cabk, junc_) and subsarcolemmal (I_Cabk,sl_) components:

$$I_{Cabk_{junc}}=F_{junc}G_{bkg}\left(V_{m}-E_{Ca_{junc}}\right),$$ (1)

$$I_{Cabk_{sl}}=F_{sl}G_{bkg}\left(V_{m}-E_{Ca_{sl}}\right),$$ (2)

$$I_{Cabk}=I_{Cabk_{junc}}+I_{Cabk_{sl}},$$ (3)

where F_junc_ = 0.11 and F_sl_ = 0.89 are constants that determine the fraction of total background current corresponding to the junctional and subsarcolemmal spaces, respectively, G_bkg_ is the maximum conductance (5.513e-4 A/F) of the channels, V_m_ is the transmembrane voltage, and E_Ca,junc_ and E_Ca,sl_ are Nernst potentials corresponding to the junctional and subsarcolemmal spaces, respectively. SR Ca^2+^ leak (J_leak_) describes the Ca^2+^ flux out of the SR:

$$J_{\mathrm{leak}}=K_{leak}\left({\left[Ca^{2+}\right]}_{SR}-{\left[Ca^{2+}\right]}_{j}\right),$$ (4)

where K_leak_ is the leak constant (5.348e-6/ms), and [Ca^2+^]_SR_ and [Ca^2+^]_j_ describe the Ca^2+^ concentrations in the SR and junctional space, respectively. In this study, we modulated G_bkg_ and K_leak_ independently and in conjunction to investigate the effects of altered sarcolemmal Ca^2+^ entry and altered SR Ca^2+^ leak, respectively.

**FIGURE 1:**
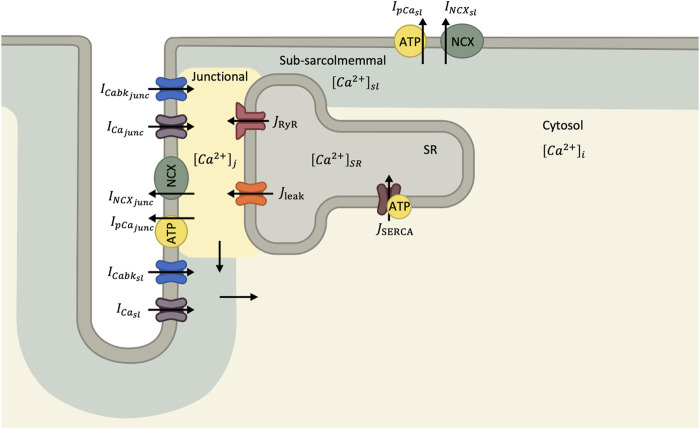
Diagram of Ca^2+^ handling components in human ventricular myocyte model (Grandi et al., 2010). In this mathematical model, the cytosol is divided into junction, sub-sarcolemmal and bulk compartments. Ca^2+^ currents through the sarcolemma enter both the junctional and sub-sarcolemmal compartments before diffusing to the bulk cytosol.

A graphical summary of the Ca^2+^ currents in the cell model is shown in Figure 1. The total Ca^2+^ current into the junctional (I_Ca,tot,junc_) and subsarcolemmal (I_Ca,tot,sl_) compartments are determined by Eqs 5, 6, respectively:

$$I_{Ca_{tot\;junc}}=I_{Ca_{junc}}+I_{Cabk_{junc}}+\;I_{pCa_{junc}}-2\cdot I_{NCX_{junc}},$$ (5)

$$I_{Ca_{tot\;sl}}=I_{Ca_{sl}}+I_{Cabk_{sl}}+\;I_{pCa_{sl}}-2\cdot I_{NCX_{sl}},$$ (6)

where I_Ca_ describes the L-type Ca^2+^ current, I_pCa_ describes the sarcolemmal Ca^2+^ pump current, and I_NCX_ describes the NCX current.

The rate of change of Ca^2+^ concentrations in the junctional compartment [Ca^2+^]_j_, subsarcolemmal compartment [Ca^2+^]_sl_, bulk cytosol [Ca^2+^]_i_, and SR [Ca^2+^]_SR_ are given by:

$$\frac{d{\left[Ca^{2+}\right]}_{j}}{dt}=-I_{Ca_{tot_{junc}}}\cdot \frac{C_{mem}}{V_{junc}\cdot 2\cdot F}+\frac{J_{Ca_{juncsl}}}{V_{junc}}\cdot \left({\left[Ca^{2+}\right]}_{sl}-{\left[Ca^{2+}\right]}_{j}\right)-J_{Ca_{B_{junction}}}+J_{RYR}\cdot \frac{V_{sr}}{V_{junc}}+J_{leak}\cdot \frac{V_{myo}}{V_{junc}},$$ (7)

$$\frac{d{\left[Ca^{2+}\right]}_{sl}}{dt}=-I_{Ca_{tot_{sl}}}\cdot \frac{C_{mem}}{V_{sl}\cdot 2\cdot F}+\frac{J_{Ca_{juncsl}}}{V_{sl}}\cdot \left({\left[Ca^{2+}\right]}_{j}-{\left[Ca^{2+}\right]}_{sl}\right)+\frac{J_{Ca_{slmyo}}}{V_{sl}}\cdot \left({\left[Ca^{2+}\right]}_{i}-{\left[Ca^{2+}\right]}_{sl}\right)-J_{Ca_{B_{sl}}},$$ (8)

$$\frac{d{\left[Ca^{2+}\right]}_{i}}{dt}=-J_{SERCA}\cdot \frac{V_{sr}}{V_{myo}}-J_{Ca_{B_{cytosol}}}+\frac{J_{Ca_{slmyo}}}{V_{myo}}\cdot \left({\left[Ca^{2+}\right]}_{sl}-{\left[Ca^{2+}\right]}_{i}\right),$$ (9)

$$\frac{d{\left[Ca^{2+}\right]}_{SR}}{dt}=J_{SERCA}-\left(J_{leak}\cdot \frac{V_{myo}}{V_{sr}}+J_{RyR}\right)-\frac{dCsqn_{b}}{dt},$$ (10)

where C_mem_ is the membrane capacitance, F is Faraday’s constant, V_junc_ is the volume of the junctional compartment, V_sl_ is the volume of the subsarcolemmal compartment, V_myo_ is the volume of the bulk cytosol, and V_sr_ is the volume of the SR. J_Ca,juncsl_ is the rate of Ca^2+^ flux from junctional to subsarcolemmal compartments and J_Ca,slmyo_ is the rate of Ca^2+^ flux from the subsarcolemmal compartment to bulk cytosol. J_RYR_ describes the SR Ca^2+^ release and J_SERCA_ describes the SR Ca^2+^ pump. Ca^2+^ buffering is described by J_Ca,B,junction_ in the junctional compartment, J_Ca,B,sl_ in the subsarcolemmal compartment, J_Ca,B,cytosol_ in the bulk cytosol, and dCsqn_b_/dt in the SR. We refer to (Grandi et al., 2010) for more details and the equations describing the mathematical model.

### Evaluating the Effects of Background and Leak Ca^2+^ Currents on Ca^2+^ Concentrations

To investigate the effects of altered background Ca^2+^ entry, we modulated G_bkg_ from 0 to 300% of its default value, 5.513e-4 A/F. To investigate the effects of altered SR Ca^2+^ leak, we modulated K_leak_ from 0 to 300% of its default value, 5.348e-6 ms^−1^. Simulation durations were 1 min to establish steady state. We analyzed the last beat. We performed sensitivity analyses for the modulation of G_bkg_ and K_leak_ independently on measurements of resting V_m_, maximum V_m_, action potential duration to 90% repolarization (APD_90_), diastolic [Ca^2+^]_i_, systolic [Ca^2+^]_i_, [Ca^2+^]_i_ amplitude, diastolic [Ca^2+^]_SR_, systolic [Ca^2+^]_SR_, and [Ca^2+^]_SR_ amplitude. Sensitivity analyses of each measured parameter were then normalized to the measurement of the default model values and fit to the quadratic equation:

$$y\left(x\right)=Ax^{2}+Bx+C,$$ (11)

where x is the fraction (%) of default G_bkg_ or K_leak_. We evaluated the factor of the quadratic term, A, the linear term, B, and the constant term, C, of this fit for relative comparisons of the effects of modulated background Ca^2+^ current or modulated SR Ca^2+^ leak. This analysis was repeated for the model ran at 1, 2, and 3 Hz electrical excitation.

We subsequently performed a dual-sensitivity analysis for the modulations of G_bkg_ and K_leak_ to understand how the two Ca^2+^ currents interact and influence Ca^2+^ handling in the cardiomyocyte together.

## Results

### Qualitative Changes in V_m_, [Ca^2+^]_i_, and [Ca^2+^]_SR_ When Background Ca^2+^ Entry is Modulated

**FIGURE 2:**
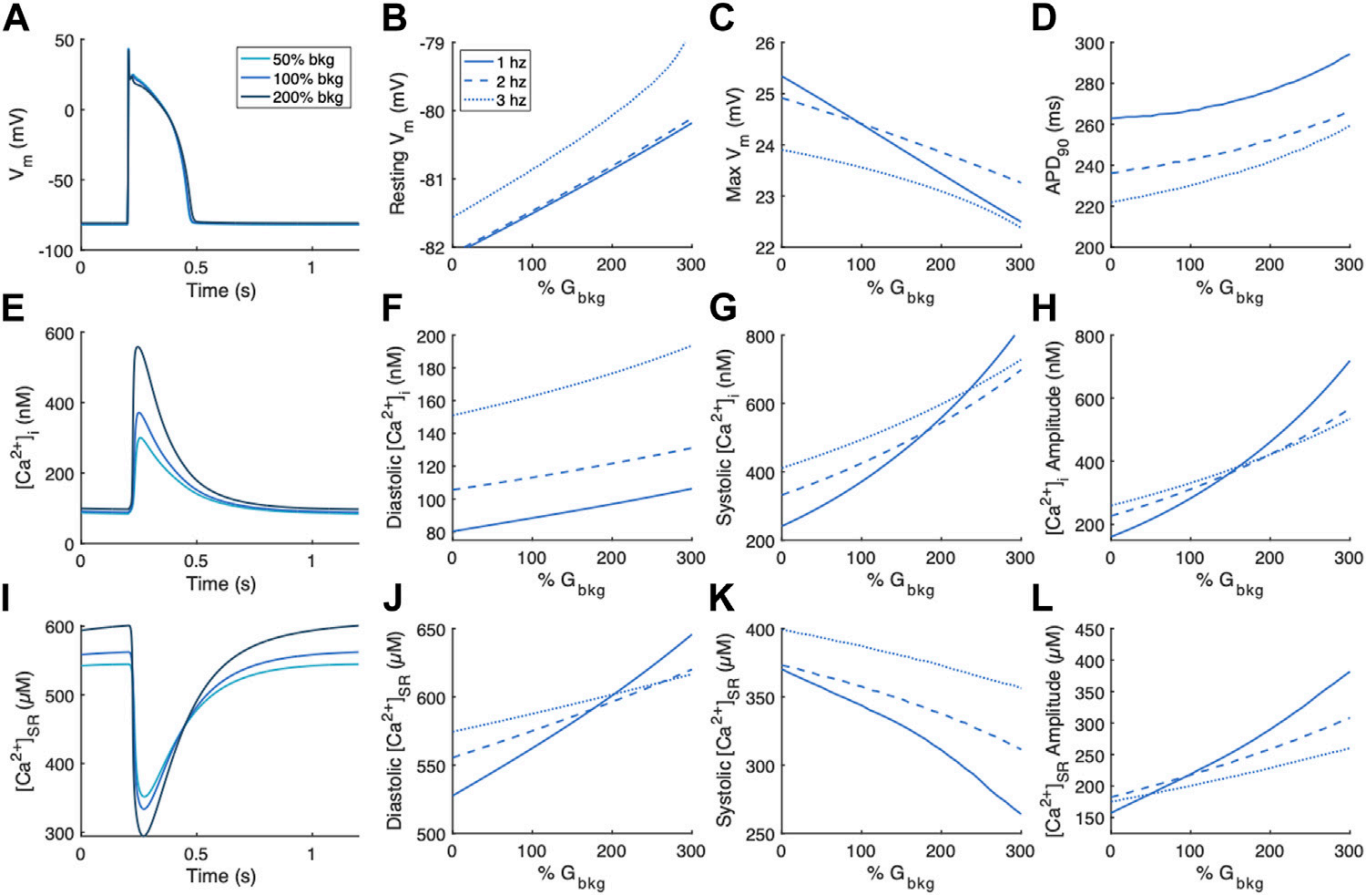
Effects of modulating the background Ca^2+^ entry current, I_Cabk,_ on V_m_, [Ca^2+^]_i_, and [Ca^2+^]_SR._ **(A)** Example action potentials for 50% and 200% G_bkg_ at 1 Hz pacing. Sensitivity analyses of modulating G_bkg_ on measurements of **(B)** resting V_m_, **(C)** maximum V_m_, and **(D)** time to 90% repolarization at 1, 2 and 3 Hz pacing. **(E)** Example [Ca^2+^]_i_ transients for 50% and 200% G_bkg_ at 1 Hz pacing. Sensitivity analyses of modulating G_bkg_ on measurements of **(F)** diastolic [Ca^2+^]_i_, **(G)** systolic [Ca^2+^]_i_, and **(H)** [Ca^2+^]_i_ amplitude at 1, 2 and 3 Hz pacing. **(I)** Example [Ca^2+^]_SR_ transients for 50% and 200% G_bkg_ at 1 Hz pacing. Sensitivity analyses of modulating G_bkg_ on measurements of **(J)** diastolic [Ca^2+^]_SR_, **(K)** systolic [Ca^2+^]_SR_, and **(L)** [Ca^2+^]_SR_ amplitude at 1, 2 and 3 Hz pacing.

We first examined the changes in V_m_, [Ca^2+^]_i_, and [Ca^2+^]_SR_ following modulation of the background Ca^2+^ entry current, I_Cabk,_ at pacing rates of 1, 2 and 3 Hz (Figure 2). Changes to features of the action potential were minimal (Figure 2A). Resting V_m_ exhibited a positive relationship with % G_bkg_ (Figure 2B), while maximum depolarization exhibited a negative relationship (Figure 2C) with increasing background Ca^2+^ current. APD_90_ showed a positive relationship, where increased background Ca^2+^ entry lengthens the time for repolarization (Figure 2D). We measured diastolic and systolic values, as well as amplitude of [Ca^2+^]_i_ transients (Figure 2E). All measurements demonstrate positive relationships with increasing background Ca^2+^ entry; the increase in systolic [Ca^2+^]_i_ (Figure 2F) is greater than the increase in diastolic [Ca^2+^]_i_ (Figure 2G), especially at the slowest pacing rate, so the amplitude increases a considerable amount (Figure 2H). We measured the same parameters of [Ca^2+^]_SR_ transients, which also demonstrate an increase in amplitude caused by increased background Ca^2+^ entry (Figures 2I,L). The release was initiated from increased diastolic [Ca^2+^]_SR_ (Figure 2J) and ended in reduced systolic [Ca^2+^]_SR_ (Figure 2K). The relationships between background Ca^2+^ entry and features of the [Ca^2+^]_SR_ transient were also augmented at the slowest pacing rate.

### Qualitative Changes in V_m_, [Ca^2+^]_i_, and [Ca^2+^]_SR_ When SR Ca^2+^ Leak is Modulated

**FIGURE 3:**
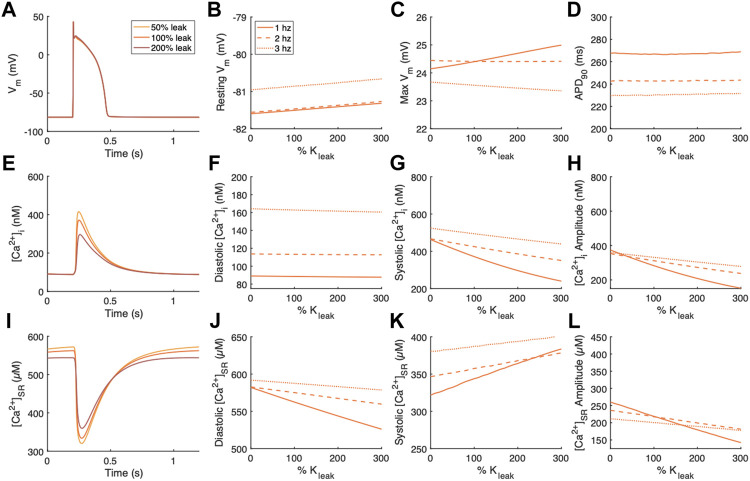
Effects of modulating the SR Ca^2+^ leak flux, J_leak,_ on V_m_, [Ca^2+^]_i_, and [Ca^2+^]_SR._ **(A)** Example action potentials for 50% and 200% K_leak_ at 1 Hz pacing. Sensitivity analyses of modulating K_leak_ on measurements of **(B)** resting V_m_, **(C)** maximum V_m_, and **(D)** time to 90% repolarization at 1, 2 and 3 Hz pacing. **(E)** Example [Ca^2+^]_i_ transients for 50% and 200% K_leak_ at 1 Hz pacing. Sensitivity analyses of modulating K_leak_ on measurements of **(F)** diastolic [Ca^2+^]_i_, **(G)** systolic [Ca^2+^]_i_, and **(H)** [Ca^2+^]_i_ amplitude at 1, 2 and 3 Hz pacing. **(I)** Example [Ca^2+^]_SR_ transients for 50% and 200% K_leak_ at 1 Hz pacing. Sensitivity analyses of modulating K_leak_ on measurements of **(J)** diastolic [Ca^2+^]_SR_, **(K)** systolic [Ca^2+^]_SR_, and **(L)** [Ca^2+^]_SR_ amplitude at 1, 2 and 3 Hz pacing.

Changes following independent modulation of the SR Ca^2+^ leak current, J_leak,_ were also examined (Figure 3). The effects of modulating K_leak_ on V_m_ were small (Figure 3A). Resting V_m_ exhibited a slight positive relationship (Figure 3B). Maximum V_m_ increased with increasing K_leak_ at 1 Hz pacing but decreased with K_leak_ at 2 and 3 Hz pacing (Figure 3C). Repolarization time measured as APD_90_ was unaltered by modulations of K_leak_ (Figure 3D). Diastolic [Ca^2+^]_i_ was also relatively unaffected by modulations of K_leak_ (Figures 3E,F). However systolic [Ca^2+^]_i_ and thus also [Ca^2+^]_i_ amplitude decreased with increasing K_leak_ (Figures 3G,H). The effect was strongest at the slowest pacing frequency of 1 Hz. Alterations in [Ca^2+^]_SR_ were also strongest at 1 Hz pacing. Diastolic [Ca^2+^]_SR_ exhibited a negative relationship with K_leak_ (Figure 3J), while systolic [Ca^2+^]_SR_ exhibits a positive relationship with K_leak_ (Figure 3K), both contributing to reduced [Ca^2+^]_SR_ amplitude with increased K_leak_ (Figure 3L).

### Summary and Comparison of Independent Modulation of Background and Leak Currents

**FIGURE 4:**
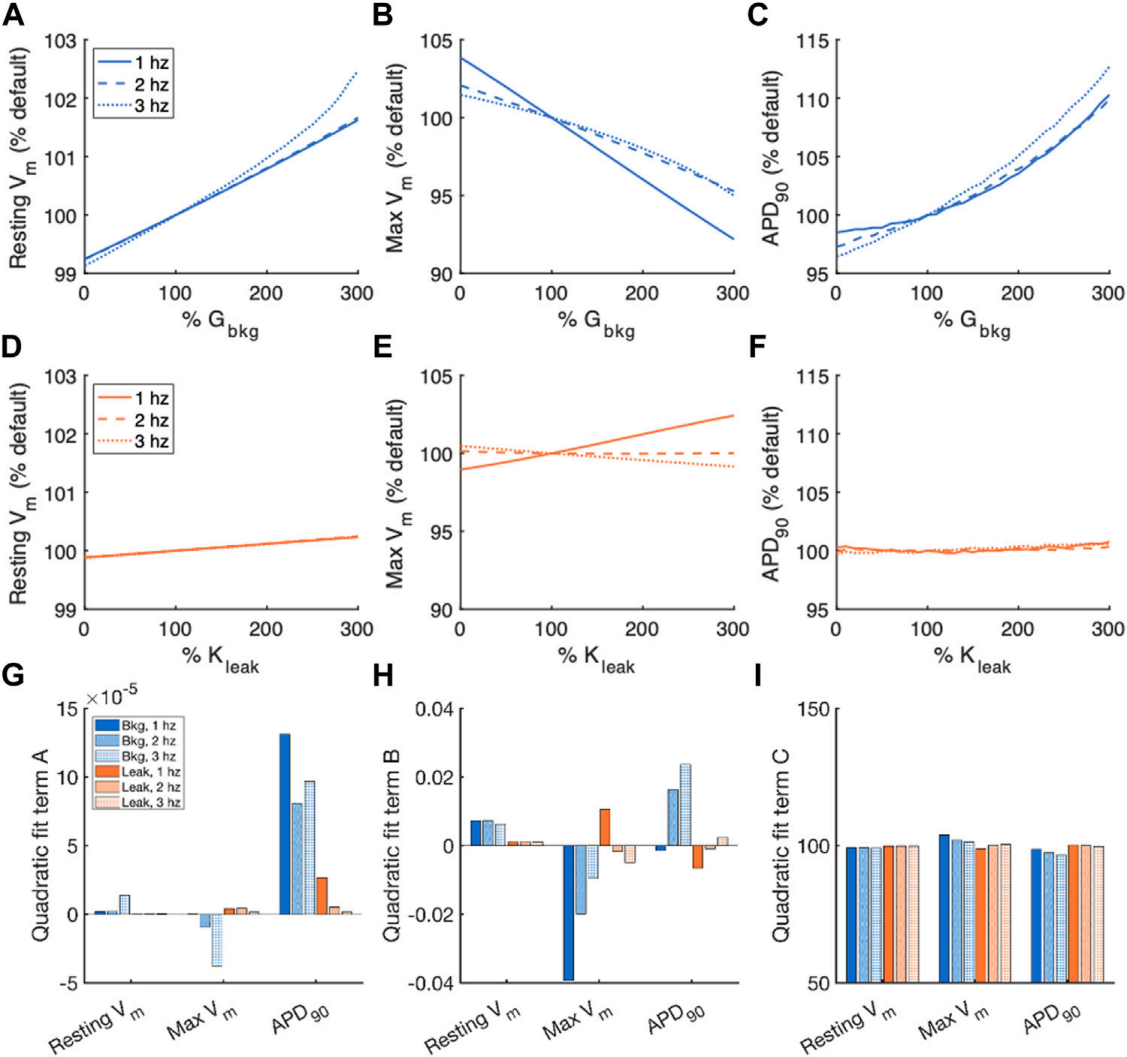
Summary of effects of modulating G_bkg_ or K_leak_ independently on V_m_. Sensitivity analyses of modulating G_bkg_ on measurements of **(A)** resting V_m_, **(B)** maximum V_m_, and **(C)** time to 90% repolarization normalized by the default model parameters. Sensitivity analyses of modulating K_leak_ on measurements of **(D)** resting V_m_, **(E)** maximum V_m_, and **(F)** time to 90% repolarization normalized by the default model parameters. Summary of the **(G)** quadratic term, **(H)** linear term and **(I)** constant term calculated by quadratic polynomial fits to the normalized sensitivity analyses in **(A–H)**.

For comparison of the effects of altered G_bkg_ or K_leak_ on features of the action potential, we normalized the measurements of resting V_m_, maximum V_m_, and APD_90_ to the default measurements from the model for the given pacing frequency (Figures 4A–F). The relationships of measured vs. modified parameter, both represented as fraction (%) vs. default values, were fit to a 2nd order polynomial model for quantification of the effects (Figures 4G–I). The quadratic term of the fit is negligible for resting V_m_ and maximum V_m_, demonstrating that these measurements exhibit primarily linear relationships with G_bkg_ and K_leak_ (Figure 4G). However, the polynomial fit of APD_90_ to G_bkg_ has a strong quadratic term, demonstrating a non-linear relationship between APD_90_ and G_bkg_ (Figure 4G). Since the relationships of resting V_m_ and maximum V_m_ are primarily linear, the linear term of the quadratic polynomial fit demonstrates the sensitivity of the measurements to changes in G_bkg_ or K_leak_ (Figure 4H). Both G_bkg_ and K_leak_ modulation have a positive relationship with resting V_m_, but the changes are negligible for K_leak_ and minimal for G_bkg_, never exceeding an increase greater than 2% of the default model’s resting V_m_. Maximum V_m_ had a strong negative linear relationship with G_bkg_ for all pacing frequencies, especially at 1 Hz pacing. Maximum V_m_ had a positive linear relationship with K_leak_ at 1 Hz pacing but negatives linear relationship for 2 and 3 Hz. The constant of the quadratic polynomial model represents the measurements for the modulated currents set to 0 (Figure 4I). These results revealed that resting V_m_ was largely unchanged by pacing rate. Maximum V_m_ was slightly increased with no I_Cabk_ and slightly reduced with no J_leak_ at 1 Hz and slightly increased with no J_leak_ at 3 Hz. APD_90_ decreased without I_Cabk_ but remained unchanged without K_leak_.

**FIGURE 5:**
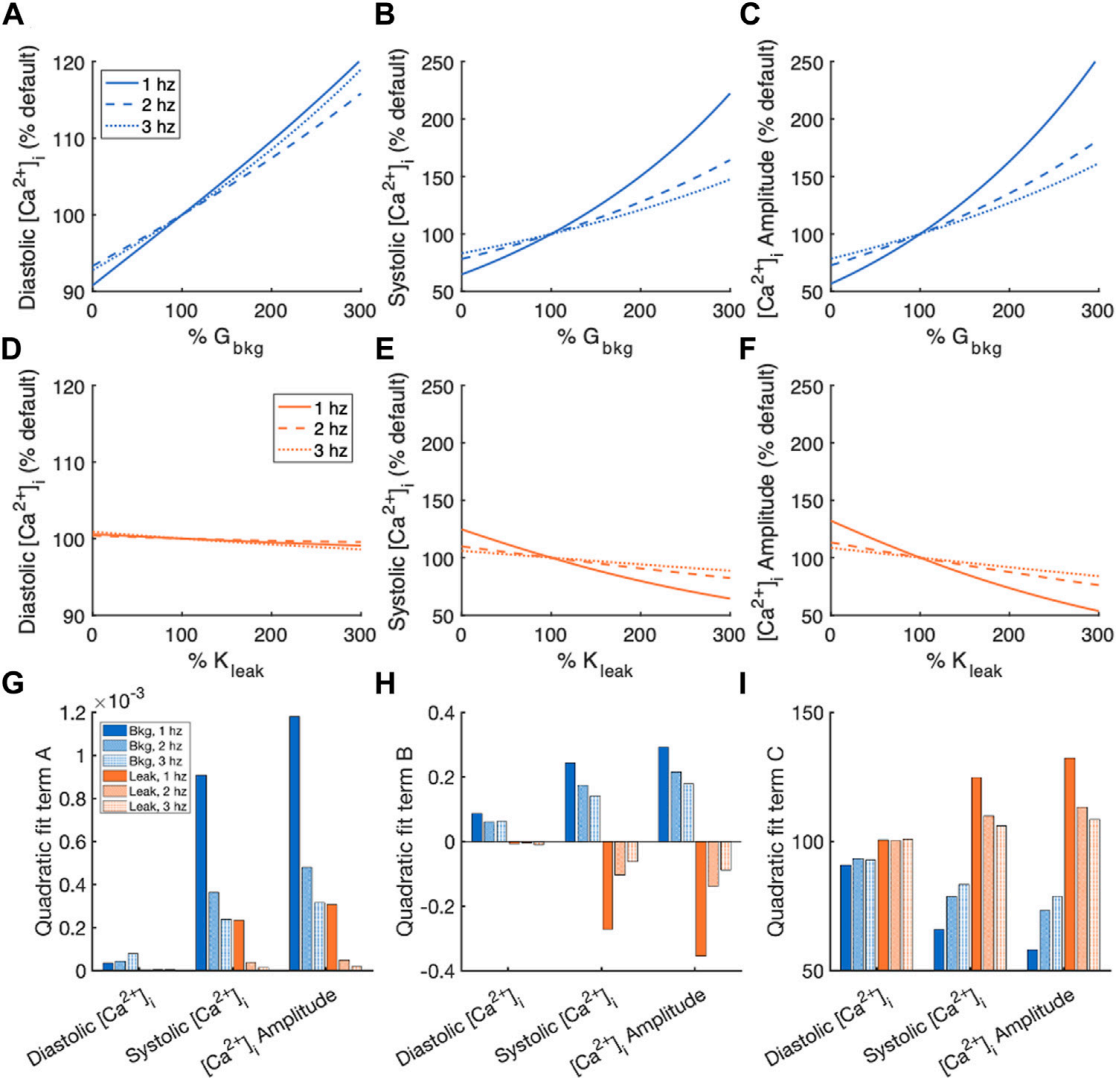
Summary of effects of modulating G_bkg_ or K_leak_ independently on [Ca^2+^]_i_. Sensitivity analyses of modulating G_bkg_ on measurements of **(A)** diastolic [Ca^2+^]_i_, **(B)** systolic [Ca^2+^]_i_, and **(C)** [Ca^2+^]_i_ amplitude normalized by the default model parameters. Sensitivity analyses of modulating K_leak_ on measurements of **(D)** diastolic [Ca^2+^]_i_, **(E)** systolic [Ca^2+^]_i_, and **(F)** [Ca^2+^]_i_ amplitude normalized by the default model parameters. Summary of the **(G)** quadratic term, **(H)** linear term and **(I)** constant term calculated by quadratic polynomial fits to the normalized sensitivity analyses in **(A–H)**.

Figure 5 contains a summary of [Ca^2+^]_i_ normalized by measurements of the default model for the sensitivity analyses of G_bkg_ and K_leak_ modulation. The relationships of measured parameter vs. modified parameter, both represented as % default values, were fit to a 2nd order polynomial model for quantification of the effects. The quadratic term indicates nonlinearity of the relationship (Figure 5G). The quadratic term of the diastolic [Ca^2+^]_i_ fits were marginal, indicating a primarily linear relationship. For both G_bkg_ and K_leak_ modulations, we noticed a nonlinearity associated with systolic [Ca^2+^]_i_ and consequently the [Ca^2+^]_i_ amplitude, with the greatest degree of nonlinearity associated with the lowest pacing frequency. The linear term of the quadratic fit showed a strong linear sensitivity of the measured parameter to changes in G_bkg_ or K_leak_ (Figure 5H). The positive sign of this term for G_bkg_ modulations for each measurement, diastolic, systolic, and amplitude, demonstrates the positive relationship of these parameters with G_bkg_, with greater slope of the relationship for systolic and amplitude measurements. The linear term for diastolic [Ca^2+^]_i_ sensitivity to K_leak_ was negative but very small, demonstrating that diastolic [Ca^2+^]_i_ exhibits negligible sensitivity to SR Ca^2+^ leak. The relationship of systolic [Ca^2+^]_i_ sensitivity to K_leak_ was negative, with the largest value for 1 Hz pacing. The same is true for [Ca^2+^]_i_ transient amplitude. The constant term of the quadratic polynomial fits corresponds to the % default if the modulated channel were 0 (Figure 5I). Elimination of I_Cabk_ resulted in a reduction of diastolic [Ca^2+^]_i,_ systolic [Ca^2+^]_i,_ and [Ca^2+^]_i_ amplitude, with the greatest reductions at 1 Hz pacing. Elimination of J_leak_ did not affect diastolic [Ca^2+^]_i,_ but results in increased systolic [Ca^2+^]_i_ and [Ca^2+^]_i_ amplitude, especially at 1 Hz pacing.

**FIGURE 6:**
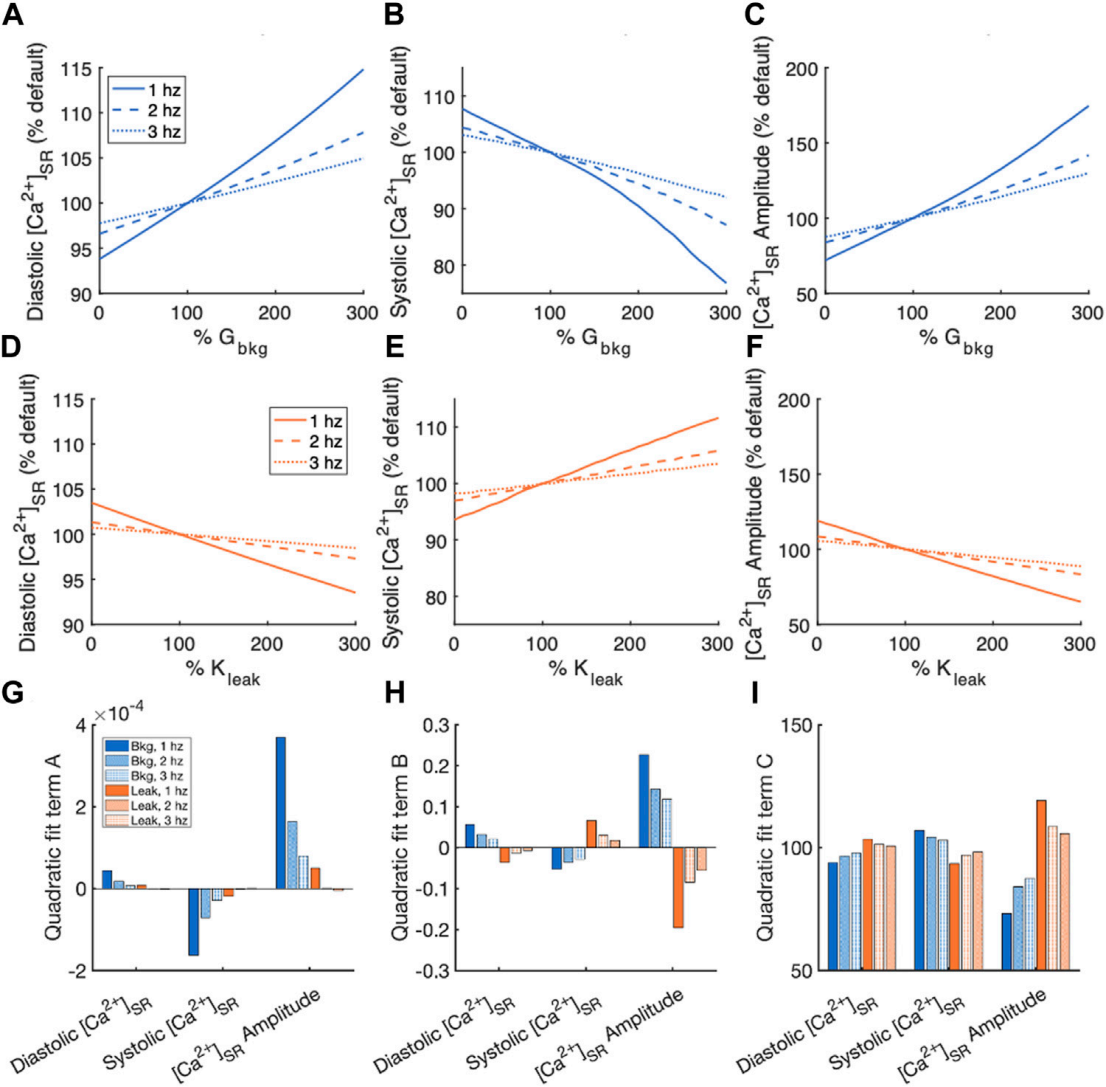
Summary of effects of modulating G_bkg_ or K_leak_ independently on [Ca^2+^]_SR_. Sensitivity analyses of modulating G_bkg_ on measurements of **(A)** diastolic [Ca^2+^]_SR_, **(B)** systolic [Ca^2+^]_SR_, and **(C)** [Ca^2+^]_SR_ amplitude normalized by the default model parameters. Sensitivity analyses of modulating K_leak_ on measurements of **(D)** diastolic [Ca^2+^]_SR_, **(E)** systolic [Ca^2+^]_SR_, and **(F)** [Ca^2+^]_SR_ amplitude normalized by the default model parameters. Summary of the **(G)** quadratic term, **(H)** linear term and **(I)** constant term calculated by quadratic polynomial fits the normalized sensitivity analyses in **(A–H)**.

Measurements of diastolic and systolic [Ca^2+^]_SR_ and [Ca^2+^]_SR_ transient amplitude, normalized by measurements of the default model, for the sensitivity analyses of G_bkg_ and K_leak_ modulations are summarized in Figure 6. Again, a 2nd order polynomial model for the normalized sensitivity analyses provided information about the relationships. The quadratic term of the fits demonstrates that systolic [Ca^2+^]_SR_ and [Ca^2+^]_SR_ transient amplitude both exhibited nonlinearity in the relationship to modulated G_bkg_ (Figure 6G). The linear term of the relationships demonstrated the best representation sensitivity of the measurements to G_bkg_ or K_leak_ modulation (Figure 6H). The sign of this term was opposite for each measured parameter for G_bkg_ vs. K_leak_ modulation but similar in amplitude. While the changes to [Ca^2+^]_SR_ transients all contributed to a positive correlation between G_bkg_ and [Ca^2+^]_SR_ transient amplitude, the opposite changes all contribute to a negative correlation between K_leak_ and [Ca^2+^]_SR_ transient amplitude. The constant term of the polynomial model, which corresponds to the % of default model measurements if the current is set to 0, showed that in the absence of I_Cabk_, diastolic [Ca^2+^]_SR_ is lower, systolic [Ca^2+^]_SR_ is higher, and [Ca^2+^]_SR_ amplitude is reduced (Figure 6I). In the absence of J_leak_, diastolic [Ca^2+^]_SR_ was slightly elevated, systolic [Ca^2+^]_SR_ was reduced, and the amplitude of [Ca^2+^]_SR_ was greater. The effects on all measurements of [Ca^2+^]_SR_ following modulation of G_bkg_ or K_leak_ were stronger at 1 Hz pacing than at faster pacing frequencies.

### Effects of Dual Modulation of Background Ca^2+^ Entry and SR Ca^2+^ Leak on Vm, [Ca^2+^]_i_, and [Ca^2+^]_SR_

**FIGURE 7:**
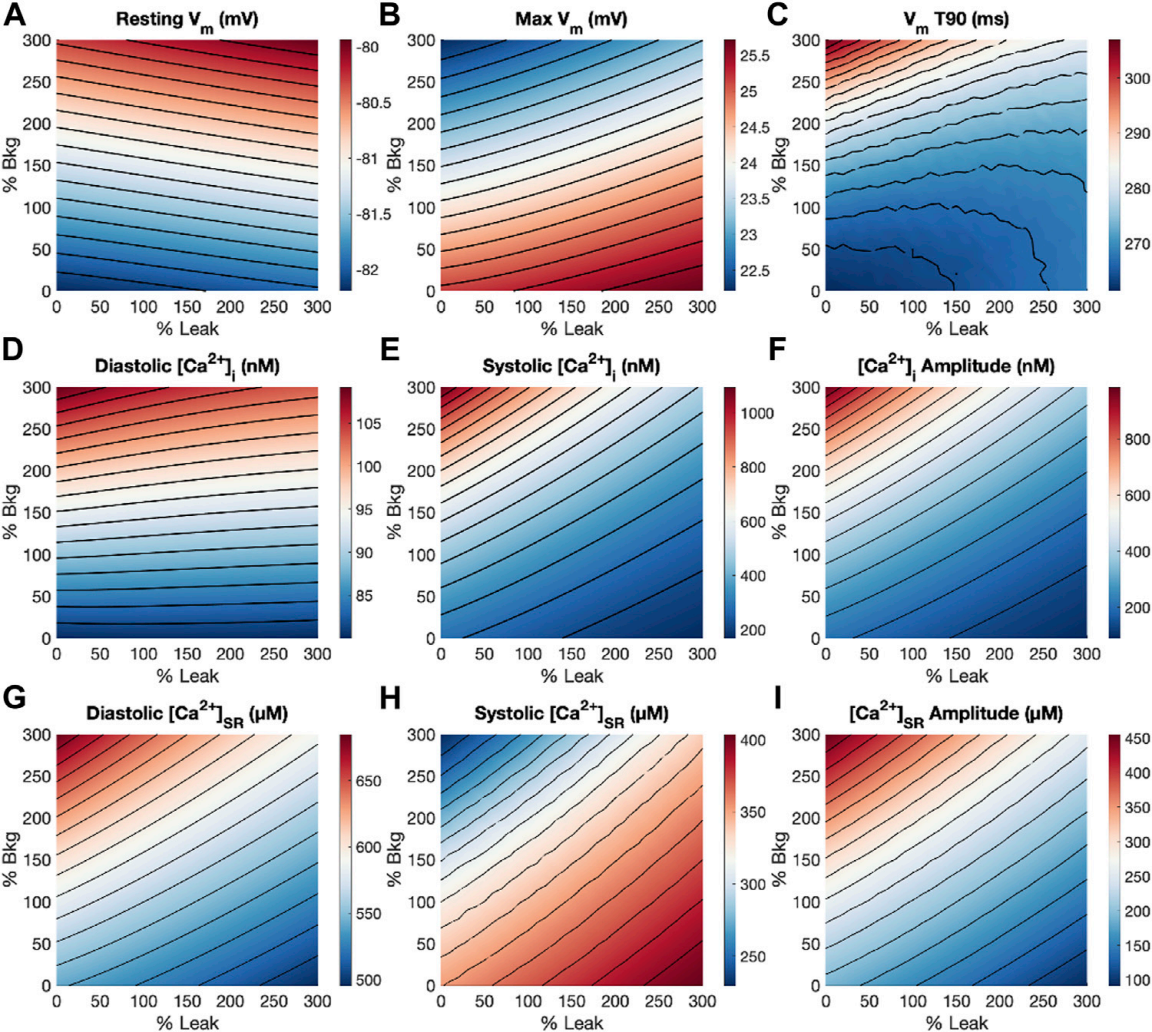
Dual-parameter sensitivity analysis of modulating G_bkg_ and K_leak_ at 1 Hz pacing. Surface plots with contour lines for measurements of **(A)** resting V_m_, **(B)** maximum V_m_, **(C)** APD_90_, **(D)** diastolic [Ca^2+^]_i_, **(E)** systolic [Ca^2+^]_i_, **(F)** [Ca^2+^]_i_ amplitude, **(G)** diastolic [Ca^2+^]_SR_, **(H)** systolic [Ca^2+^]_SR_, and **(I)** [Ca^2+^]_SR_ amplitude.

With a thorough understanding of how G_bkg_ and K_leak_ modulations independently affect features of the action potential, [Ca^2+^]_i_ and [Ca^2+^]_SR_ transients, we subsequently performed a dual-parameter sensitivity analysis of G_bkg_ and K_leak_ together for 1 Hz pacing (Figure 7). Resting V_m_ is affected minimally by G_bkg_, and negligibly by K_leak_, apparent by the nearly horizontal contour lines (Figure 7A). Both increasing G_bkg_ and increasing K_leak_ resulted in increased V_m_, but increasing G_bkg_ makes a larger contribution. Maximum V_m_ was modulated in opposite directions by G_bkg_ and K_leak_ (Figure 7B). Increasing G_bkg_ reduces maximum V_m_ while increasing K_leak_ increased maximum V_m_, but G_bkg_ is the slightly more dominant effect. APD_90_ experienced the greatest increase at large increases in G_bkg_ and small K_leak_ (Figure 7C). Diastolic [Ca^2+^]_i_ was primarily affected by a positive relationship to G_bkg_, while K_leak_ appeared to make no contribution (Figure 7D). Interestingly, for all other [Ca^2+^]_i_ and [Ca^2+^]_SR_ measurements, the opposing effects of G_bkg_ and K_leak_ appeared to balance with a very similar linear relationship (Figures 7E–I). The contour lines of the dual-parameter sensitivity analyses for systolic [Ca^2+^]_i_, [Ca^2+^]_i_ amplitude, diastolic and systolic [Ca^2+^]_SR_ and [Ca^2+^]_SR_ amplitude all exhibited strikingly similar slopes.

**FIGURE 8:**
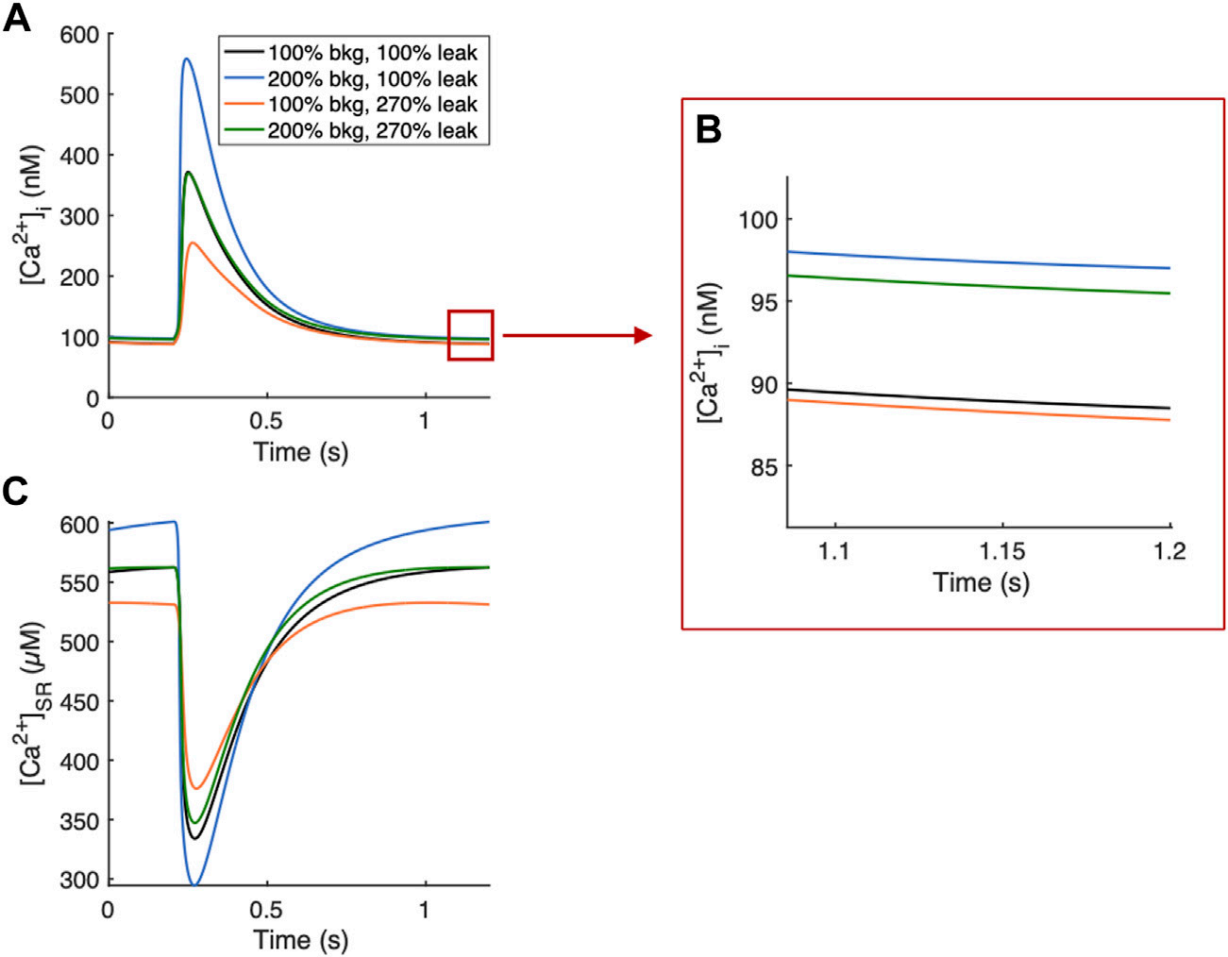
Example Ca^2+^ transients of balanced background Ca^2+^ entry and SR Ca^2+^ leak. **(A)** 200% G_bkg_ and 270% K_leak_ balance their opposing modulations on systolic [Ca^2+^]_i_ while increasing diastolic [Ca^2+^]_i_ by 7.9%. **(B)** Zoomed-in region demonstrating 7.9% increase in diastolic [Ca^2+^]_i_ from default values (black line). **(C)** 200% G_bkg_ and 270% K_leak_ balance opposing effects on SR load.

If both background and leak currents were modulated along this relationship, measured parameters remained relatively unchanged and only diastolic [Ca^2+^]_i_ increased. An example of balanced background Ca^2+^ entry and SR Ca^2+^ leak demonstrates that a G_bkg_ value 200% of default and K_leak_ value of 270% default provided a balanced effect and canceled out the opposing modulations on systolic [Ca^2+^]_i_ and diastolic [Ca^2+^]_SR_ load, while diastolic [Ca^2+^]_i_ was elevated 7.9% (Figure 8).

## Discussion

In this study, we evaluated the contributions of background Ca^2+^ entry and SR Ca^2+^ leak to V_m_ and Ca^2+^ concentrations in the SR and cytosol in cardiomyocytes *in silico*. Our investigations shed light on the differential effects of background and leak Ca^2+^ currents in physiology, and also provide insight into their contributions to disease development due to Ca^2+^ dysfunction. Below, we discussed background Ca^2+^ entry as a mechanism to positively modulate Ca^2+^ entry and SR Ca^2+^ leak as a critical balancing mechanism to maintain homeostasis.

### Background Ca^2+^ Entry Positively Modulates Ca^2+^ Concentrations

Our results show that background Ca^2+^ entry has a positive relationship with diastolic [Ca^2+^]_i_ and [Ca^2+^]_SR_, and the amplitude of their transients (Figure 2). The small increase in diastolic [Ca^2+^]_i_ is the direct result of an increase in background Ca^2+^ entry, and the increase in [Ca^2+^]_SR_ follows as SERCA responds to pump the extra Ca^2+^ into the SR. The small increase in diastolic [Ca^2+^]_i_ amplifies Ca^2+^-induced-Ca^2+^ release through RyRs, explaining the increased transient amplitudes. This supports the physiological role of background Ca^2+^ entry in increasing Ca^2+^ concentrations. These results replicate prior findings for TRP family members suggested as Ca^2+^ entry channels. For example, we provided evidence for TRPC6 as background Ca^2+^ entry in neonatal rat ventricular myocytes (Ahmad et al., 2020). Overexpression of TRPC6 contributes to both elevated [Ca^2+^]_i_ and [Ca^2+^]_SR_. TRPC3 and TRPC6 have also both been implicated in the stress-induced slow increase in [Ca^2+^]_i_ and increased [Ca^2+^]_i_ transients contributing to the SFR (Seo et al., 2014; Yamaguchi et al., 2017; Yamaguchi et al., 2018). These studies highlight one potential role for Ca^2+^ entry in strained myocytes. Many of the candidates suggested as background Ca^2+^ entry channels are known to be modulated by stretch (Inoue et al., 2009; Reed et al., 2014; Peyronnet et al., 2016), so strained myocytes would exhibit an increase in background Ca^2+^ entry, leading to elevated diastolic levels and larger transients. This mechanism likely contributes to the SFR, increased contractile force following sustained stretch.

### SR Ca^2+^ Leak Negatively Modulates Ca^2+^ Concentrations as a Balancing Mechanism

SR Ca^2+^ leak had only a marginal effect on diastolic [Ca^2+^]_i_ but reduced [Ca^2+^]_SR_ and the amplitude of Ca^2+^ transients (Figure 3). In general, modulating SR Ca^2+^ leak had the opposite effects of background Ca^2+^ entry, except for the weak effect on diastolic [Ca^2+^]_i_. The leaked Ca^2+^ is removed from the cytosol effectively by NCX, which is why it does not affect free cytosolic levels but reduces SR Ca^2+^. On its own, this complicates the understanding of the physiological role of SR Ca^2+^ leak and the purpose of reduced SR load. When considering the dual-parameter sensitivity analysis, it became however evident that while background Ca^2+^ entry responds to increased needs with increased Ca^2+^, SR Ca^2+^ leak likely functions as a critical balancing component to regulate SR stores and maintain Ca^2+^ homeostasis. The combined effects of increasing both background Ca^2+^ entry and SR Ca^2+^ leak exhibit a linear relationship, represented by the contour lines of the dual sensitivity plot for systolic [Ca^2+^]_i_, [Ca^2+^]_i_ amplitude, diastolic and systolic [Ca^2+^]_SR_ and [Ca^2+^]_SR_ amplitude (Figure 7). If both background and leak currents are modulated along this relationship, measured parameters remain relatively unchanged and only diastolic [Ca^2+^]_i_ increases. Examples of balanced background Ca^2+^ entry and SR Ca^2+^ leak in Figure 8 demonstrate that modulations in G_bkg_ and K_leak_ can be balanced in a way to cancel out the large opposing effects they have on Ca^2+^ transient amplitudes (Figure 8). This balancing mechanism of SR Ca^2+^ leak could be critical to prevent Ca^2+^ overload in the cell. Leak in the form of RyR sparks has been demonstrated as SR load regulator to prevent overload, with a steep dependency on [Ca^2+^]_SR_ (Shannon et al., 2002). However, large Ca^2+^ release events through RyR sparks increase sensitivity for arrhythmia (George, 2008). RyR sparks are most prevalent at high [Ca^2+^]_SR_, but non-spark RyR leak and non-RyR leak do not appear to exhibit the same steep dependency on [Ca^2+^]_SR_ and therefore might function differently. Thus, a mechanism of non-RyR leak may be to regulate compartmental Ca^2+^ before the cells become overloaded, and RyR sparks increase as a more extreme measure.

Like channels involved in background Ca^2+^ entry in cardiomyocytes, candidates for SR Ca^2+^ leak also include members of the TRP family and were suggested to be mechano-modulated (Inoue et al., 2009; Reed et al., 2014; Peyronnet et al., 2016). This indicates that the background and leak Ca^2+^ currents could be modulated in conjunction. Non-RyR leak that can be modulated by stretch may provide a more moderate and steady regulation on a beat-by-beat basis in conjunction with background Ca^2+^ entry modulated by stretch. Recently we demonstrated that TRPC1 constitutes an SR Ca^2+^ leak channel, and its overexpression resulted in decreased SR Ca^2+^ load (Hu et al., 2020). TRPC1 channels are suggested to be modulated by stretch, indicating that the reduction in SR Ca^2+^ load could be a regulatory mechanism to match increased background Ca^2+^ entry through, e.g., TRPC6 channels. We speculate that background Ca^2+^ entry and SR Ca^2+^ leak fulfill a critical homeostatic function in the modulation of Ca^2+^ concentrations throughout the cardiomyocyte in response to strain.

### Background and Leak Ca^2+^ Currents May Contribute to Hypertrophy and HF Under Chronic Pressure Overload

Both background Ca^2+^ entry and SR Ca^2+^ leak through TRP channels are likely to be modulated by cardiomyocyte strain (Inoue et al., 2009; Reed et al., 2014; Peyronnet et al., 2016). Under chronic pressure overload conditions, strain-modulation of TRPC channels could increase background Ca^2+^ entry and SR Ca^2+^ leak, and thus dysregulate Ca^2+^. Cardiac disease is perpetuated by Ca^2+^ dysregulation, and a stray from its homeostatic balance. Some of the suggested ion channels for these Ca^2+^ currents were found to be upregulated in models of cardiac disease, suggesting a role in pathogenesis (Ahmad et al., 2017; Hof et al., 2019). Diastolic [Ca^2+^]_i_ is elevated in HF causing diastolic dysfunction (Eisner et al., 2020). Elevated background Ca^2+^ entry could be a contributing factor. Two different models of HF with preserved ejection fraction (HFpEF) display increases in both diastolic and systolic [Ca^2+^]_i_ (Curl et al., 2018; Rouhana et al., 2019). A hypothesis is that a major difference in Ca^2+^ handling between HFpEF and HF with reduced ejection fraction (HFrEF) is preserved [Ca^2+^]_SR_ in HFpEF vs. reduced [Ca^2+^]_SR_ in HFrEF (Eisner et al., 2020). The decreased SR Ca^2+^ content contributes largely to the decrease in systolic [Ca^2+^]_i_ and contractile dysfunction (Bers, 2006). Based on the demonstration of a balancing mechanism between background Ca^2+^ entry and SR Ca^2+^ leak in this study, it is reasonable to speculate a difference between maintenance of this balance in HFpEF vs. a stray from this balanced relationship towards overcompensated leak in HFrEF. In addition to reducing SR Ca^2+^ available for release, causing systolic dysfunction, increased SR Ca^2+^ leak can be problematic, e.g., triggering arrhythmias and being energetically costly due to increased use of ATP to repump Ca^2+^ (Bers, 2014). Understanding the balance of background and leak Ca^2+^ currents in cardiomyocytes and how they affect Ca^2+^ homeostasis and remodeling in disease will be critical to develop effective drug therapies targeting Ca^2+^ channels.

### Background and Leak Ca^2+^ Currents are More Effective at Modulating Ca^2+^ at Lower Frequency Pacing

In this study, we observed the well-established frequency dependency of Ca^2+^ transients. Increasing the rate of stimulation increases diastolic [Ca^2+^]_i_ in isolated myocytes (Frampton et al., 1991; Antoons et al., 2002; Dibb et al., 2007; Horváth et al., 2017; Sankaranarayanan et al., 2017). Background Ca^2+^ entry and SR Ca^2+^ leak also both exhibit a frequency effect. The parameters we measured are all more sensitive to modulations of the Ca^2+^ currents at slower pacing rates than at faster pacing rates (Figures 5, 6). The sensitivity of each measured [Ca^2+^]_i_ and [Ca^2+^]_SR_ parameter to G_bkg_ and K_leak_ is greatest in amplitude for 1 Hz pacing. An explanation is that at slower pacing rates, the background and leak currents have relatively more time to contribute to the total Ca^2+^ flux per beat vs. the voltage-gated ion channels that open during the action potential and are closed at rest.

### Modulation of I_Cabk_ and J_leak_ has Marginal Effects on Action Potentials

Modulating K_leak_ had negligible effects on the action potential for any pacing frequency (Figure 4). For the values of K_leak_ tested, we found that the SR Ca^2+^ leak flux does not significantly contribute to sarcolemmal electrophysiology. Modulating G_bkg_ has marginal effects on features of action potentials (Figure 4). While G_bkg_ positively correlates with increased resting V_m_, an increase to 300% G_bkg_ only resulted in <2% change from basal resting V_m_ . This minimal change in resting potential is unlikely to be functionally relevant. An increase to 300% G_bkg_ also reduces maximum depolarization by 7% for 1 Hz pacing. The largest effect is an increase in action potential duration (APD90) by around 10% for maximal G_bkg_ modulation. It has also been shown that APD prolongation leads to increased Ca^2+^ (Bouchard et al., 1995), suggesting a positive feedback loop for electrical and Ca^2+^ signaling. Another important note is that APD increase is known to be inotropic, e.g., in rat ventricular myocytes (Bouchard et al., 1995). This indicates another mechanism for the contribution of background Ca^2+^ entry to contractility. Conversely, prolonged APD can induce torsades de pointes tachycardia, leading to life-threatening ventricular fibrillation (Roden and Hoffman, 1985; Ravens and Cerbai, 2008).

### Limitations

Mathematical modeling of cellular electrophysiology provides a valuable resource for studying how aspects of cellular physiology interact and affect one another. It provides a means to investigate questions that cannot be easily answered *in vivo*. However, there are also caveats of mathematical modeling that should be considered. It should be noted that the definitions of I_Cabk_ and J_leak_ used in this model are general simplifications and meant to reproduce poorly defined currents. The equations lack specific gating conditions of the currents. The current equations were not parameterized to match experimental data which is only incompletely characterized in human ventricular myocytes. Instead, the current equations are adjusted such that the model reproduces overall physiological action potentials and calcium transients. This is an important consideration, since the magnitudes of these currents could be largely different in living cells. Thus, interpreting the results of this study should focus on the qualitative trends. As the specific ion channels that contribute to Ca^2+^ entry and leak are identified and characterized, future work can aim to refine the current definitions and provide detailed current models to replace the general simplifications of I_Cabk_ and J_leak_.

In a similar way, other ion currents in the model are not fully defined. For example, some K^+^ channels and isoforms of the Na^+^/K^+^-ATPase are modulated by localized Ca^2+^ concentrations (Tian and Xie, 2008; Weisbrod, 2020). However, the model does not include any Ca^2+^-dependent terms in the definitions of these currents. The inclusion of these interactions may alter the effects we see on V_m_ in this study. Future work could address this limitation.

## Data Availability

The original contributions presented in the study are included in the article/Supplementary Material, further inquiries can be directed to the corresponding author.
